# New endoperoxides highly active in vivo and in vitro against artemisinin-resistant *Plasmodium falciparum*

**DOI:** 10.1186/s12936-018-2281-x

**Published:** 2018-04-03

**Authors:** Lis Lobo, Lília I. L. Cabral, Maria Inês Sena, Bruno Guerreiro, António Sebastião Rodrigues, Valter Ferreira de Andrade-Neto, Maria L. S. Cristiano, Fatima Nogueira

**Affiliations:** 1 Global Health and Tropical Medicine, GHTM, Unidade de Ensino e Investigação de Parasitologia Médica, Instituto de Higiene e Medicina Tropical, IHMT, Universidade Nova de Lisboa, UNL, Rua da Junqueira no 100, 1349-008 Lisbon, Portugal; 2 Centre of Marine Sciences, CCMAR, Universidade do Algarve, UAlg, Campus de Gambelas, 8005-139 Faro, Portugal; 3 Departmento de Química e Farmácia, Faculdade de Ciências e Tecnologia, FCT, Universidade do Algarve, Faro, Portugal; 4 Centre for Toxicogenomics and Human Health, Genetics, Oncology and Human Toxicology, Nova Medical School, Lisbon, Portugal; 5 Laboratório de Biologia da Malária e Toxoplasmose, Departamento de Microbiologia e Parasitologia, Universidade Federal do Rio Grande do Norte, Natal, Rio Grande do Norte Brazil

**Keywords:** *Plasmodium falciparum*, Antimalarial drug resistance, Trioxolane–tetrazole conjugates, Tetraoxane–tetrazole conjugates, In vivo antimalarial activity

## Abstract

**Background:**

The emergence and spread of *Plasmodium falciparum* resistance to artemisinin-based combination therapy in Southeast Asia prompted the need to develop new endoperoxide-type drugs.

**Methods:**

A chemically diverse library of endoperoxides was designed and synthesized. The compounds were screened for in vitro and in vivo anti-malarial activity using, respectively, the SYBR Green I assay and a mouse model. Ring survival and mature stage survival assays were performed against artemisinin-resistant and artemisinin-sensitive *P. falciparum* strains. Cytotoxicity was evaluated against mammalian cell lines V79 and HepG2, using the MTT assay.

**Results:**

The synthesis and anti-malarial activity of 21 new endoperoxide-derived compounds is reported, where the peroxide pharmacophore is part of a trioxolane (ozonide) or a tetraoxane moiety, flanked by adamantane and a substituted cyclohexyl ring. Eight compounds exhibited sub-micromolar anti-malarial activity (IC_50_ 0.3–71.1 nM), no cross-resistance with artemisinin or quinolone derivatives and negligible cytotoxicity towards mammalian cells. From these, six produced ring stage survival < 1% against the resistant strain IPC5202 and three of them totally suppressed *Plasmodium berghei* parasitaemia in mice after oral administration.

**Conclusion:**

The investigated, trioxolane–tetrazole conjugates LC131 and LC136 emerged as potential anti-malarial candidates; they show negligible toxicity towards mammalian cells, ability to kill intra-erythrocytic asexual stages of artemisinin-resistant *P. falciparum* and capacity to totally suppress *P. berghei* parasitaemia in mice.

**Electronic supplementary material:**

The online version of this article (10.1186/s12936-018-2281-x) contains supplementary material, which is available to authorized users.

## Background

Between 2000 and 2015, malaria incidence rates fell by 37% and malaria mortality by 60%. These achievements can be largely attributed to the implementation of the artemisinin (ART)-based combination therapy (ACT) as first-line treatment of *Plasmodium falciparum* malaria in endemic countries [1]. Artemisinin and its semisynthetic derivatives (ARTs), e.g. artesunate (ATN) and dihydroartemisinin (DHA), display a much better pharmacologic profile than other anti-malarials in use and nowadays world-wide *P. falciparum* malaria treatment relies on ACT, although resistance to ARTs is now evident in several Southeast Asian countries [2–5]. Currently, reports of clinical failure of ARTs in areas coincident with partner drug resistance are increasing [6, 7]. Resistance to ARTs has been associated with mutations in the Kelch protein, K13 (PF3D7_1343700) and manifested as delayed parasite clearance [8].

Another relevant issue related to the widespread application of artemisinins (ARTs) is the difficulty in maintaining the drug supply; artemisinin is extracted from *Artemisia annua* in low yield and ART-derivatives are obtained by a long and expensive semi-synthesis process [9]. These circumstances pushed efforts towards the development of a next generation of potent anti-malarial endoperoxides, equally effective against ART-susceptible and -resistant strains of *P. falciparum,* as well as safer and cheaper than ARTs. Synthetic trioxolanes [10] and tetraoxanes [11] are particularly promising in this context, exhibiting anti-malarial activity similar to ARTs. Amenable synthetic routes have been developed to both compound classes, enabling the preparation of chemically diverse libraries of analogues for further structure–activity relationship analysis, selection of leads, optimization and development into anti-malarial drugs or drug-candidates. This approach has yielded ozonides OZ277 (arterolane) [12] and OZ439 (artefenomel) [13], but recent studies reported that OZ277 and OZ439 are compromised by the presence of K13 mutations due to potential cross-resistance with DHA [13–15].

In a recent study, three synthetic trioxolanes were tested in vivo against a mouse model infected with artemisinin resistant parasites and the compounds showed high efficacy in clearing the infection [16]. These results inspired the expansion of the library of compounds and further investigation of their efficacy against artemisinin-resistant (ART-R) *P. falciparum* strains.

The synthesis and anti-malarial activity of an endoperoxide-type library of compounds (trioxolanes and tetraoxanes) is reported. These can be easily synthesized from relatively cheap starting materials (preparation of the 21 compounds requires from 3 to 6 synthetic steps, depending on the cyclohexyl substitution).

## Methods

### Synthesis

The procedures for the preparation of the trioxolane- and tetraoxane-derived compounds were developed by us or adapted from the literature [17]. Details for the synthesis and chemical characterization of the 21 peroxide-type compounds and their precursors are included as Additional file 1.

### Evaluation of the cytotoxicity against mammalian cells

Cytotoxicity was assessed on the mammalian cell lines HepG2-A16 (human hepatoma) and V79 (Chinese hamster lung), using a MTT based assay, as previously described [18]. Tests were conducted in triplicate for each compound, at a range of concentrations (1 mM–1.372 μM), and with culture media containing 0.5% DMSO (no drug). V79 and HepG2 cell lines were incubated for 24 and 48 h, respectively (media changed at 24 h intervals). Absorbance was read at 570 nm on a multi-mode microplate reader (Triad, Dynex Technologies), to produce a log dose-dependence curve. The LD_50_ value for each compound was estimated by non-linear interpolation of the dose-dependence curve (GraphPad Software).

### Parasite cultivation

Laboratory-adapted *P. falciparum* 3D7 (chloroquine and mefloquine sensitive), Dd2 (chloroquine-resistant, mefloquine-resistant), IPC5202 (MRA-1240, MR4, ATCC^®^ Manassas Virginia) and IPC4912 (MRA-1241, MR4, ATCC^®^ Manassas Virginia) (artemisinin-resistant *P. falciparum*) were continuously cultured and sorbitol synchronized, as previously described [19].

### IC_50_ determination

Staging and parasitaemia were determined by light microscopy of Giemsa-stained thin blood smears. Anti-malarial activity was determined using the SYBR Green I assay, as previously described [20]. Briefly, early ring stage parasites (> 80% of rings) were challenged with a 1:3 serial dilution of each compound, in concentrations ranging from 10,000–0.169 nM. Fluorescence intensity was measured with a multi-mode microplate reader (Triad, Dynex Technologies), with excitation and emission wavelengths of 485 and 535 nm, respectively, and analysed by nonlinear regression using GraphPad Prism to determine IC_50_ values.

### Ring (RSA) and mature-stage (MSA) survival assay

Ring-stage survival assays (RSA) were carried out as described in [21], with modifications. Parasite cultures were synchronized once, with 5% sorbitol, and in the next reinvasion cycle, twice, with a 6 h interval. Parasites at the ring-stage (< 10 h) or mature-stage (> 36 h) post-invasion (1% parasitaemia and 2% haematocrit) were exposed during 6 h to 700 nM dihydroartemisinin (DHA) or to ≅ 10 × IC_50_ of each of the tested compounds. Then, plates were centrifuged and supernatant replaced by drug-free culture media. After additional incubation during 66 h for ring-stage, and 42 h for mature-stage, susceptibility was assessed microscopically on Giemsa-stained thin blood smears by estimating the percentage of viable parasites that developed into a second generation of rings or trophozoites.

### In vivo anti-malarial assays

In vivo tests were performed following the Guidelines for Ethical Conduct in The Care and Use of Animals of the Federal University of Rio Grande do Norte—Brazil (CEUA/UFRN/46/2013). Evaluation of anti-malarial activity in vivo was carried out using the Peters’ 4-day suppressive test [22], with modifications, as previously described [23–25]. Swiss albino mice were infected with *Plasmodium berghei* NK65 and randomly allocated to groups of five animals. Animals were treated with 50 mg/kg/day of each of the tested compounds (dissolved in PBS containing 1% DMSO), administrated orally, for four consecutive days. The control group was treated with a solution of PBS/1% DMSO. On days 5 (D5), 7 (D7) and 10 (D10) after parasite inoculation, tail blood smears were Giemsa-stained and examined microscopically to estimate parasitaemia. Acute toxicity testing was carried out to determine adverse effects due to compounds (including weight loss, hair appearance, skin wounds and behavioural changes), and recorded daily [26].

## Results

### Synthesis

**Fig. 1:**
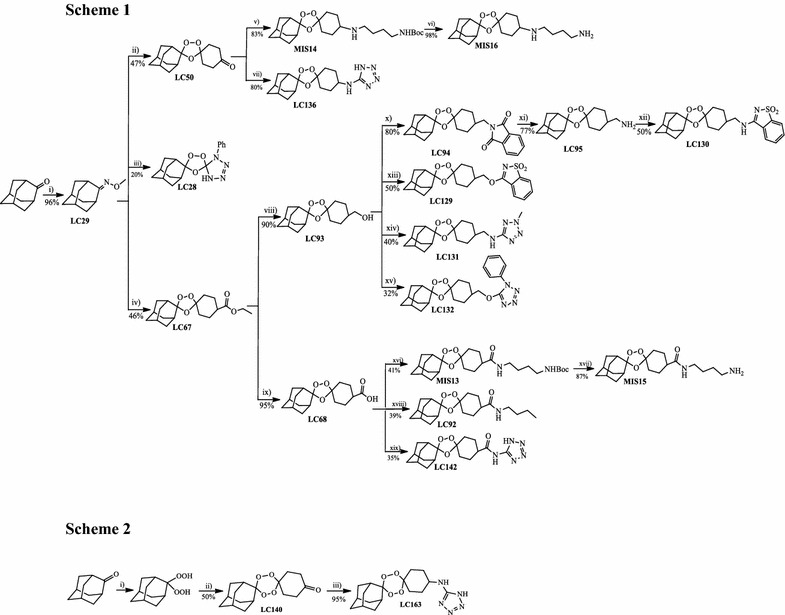
Structural representations of the trioxolanes, tetraoxanes and derivatives of artemisinin investigated in this work. Scheme 1: Representation of the synthetic routes to the trioxolanes prepared; reagents and conditions: (i) Pyridine, MeONH2, MeOH, r.t; (ii) 1,4-Cyclohexane, O3, DCM/pentane, − 78 °C; (iii) LC133, O3, DCM/pentane, − 78 °C; (iv) Ethyl 4-oxocyclohexanecarboxylate, O3, DCM/pentane, − 78 ºC; (v) LC64, AcOH, DCE, NaBH(OAc)3, r.t.; (vi) Trichloroacetic acid, DCM, H2O, r.t.; (vii) 5-Aminotetrazole, DCE, AcOH, NaBH(OAc)3, r.t.; (viii) LiBH4, Et2O, LiBH(Et)3, r.t.; (vi) KOH (3M), MeOH, 60 °C; (x) Phthalimide, Ph3P, DIAD, THF, 0ºC; (xi) Hydrazine hydrate, chloroform/MeOH, 60 °C; (xii) 3-Chloro-1,2-benzisothiazole-1,1-dioxide, THF, 60 °C; (xiii) 3-Chloro-1,2-benzisothiazole-1,1-dioxide, TEA, toluene, 45 °C; (xiv) 2-Methyl-2H-tetrazole-5-amine, TEA, mesyl chloride, THF, 60 ºC; (xv) 5-Chloro-1-phenyl-tetrazole, TEA, mesyl chloride, THF, 60 °C; (xvi) tert-Butyl(4-aminobutyl)carbamate, EDC, HOBt, N-methylmorpholine, DCM, r.t.; (xvii) Trichloroacetic acid, DCM, H2O, r.t.; (xviii) Butylamine, EDC, HOBt, N-Methylmorpholine, DCM, r.t.; (ix) 1-Aminobutane, EDC, HOBt, N-Methylmorpholine, DCM, r.t.; Scheme 2: Representation of the synthetic route to tetraoxanes LC140 and LC163; reagents and conditions: (i) HCO2H, CH3CN, H2O2 50%, 0 °C; (ii) 1,4-Cyclohexanone, DCM, HBF4, 0 °C; (iii) 5-Aminotetrazole, DCE, AcOH, NaBH(OAc)3, r.t.

A representative library of 21 compounds with potential antiplasmodial activity was designed and synthesized. All compounds proposed include a peroxide-type pharmacophore (trioxolane- or tetraoxane-based) flanked by adamantane and a suitably functionalized cyclohexyl ring. The endoperoxides LC50, LC67 and LC140 were used as intermediates for the preparation of other compounds, altering the cyclohexyl substitution. The synthesis of the trioxolane–tetrazole, tetraoxane-tetrazole and trioxolane–saccharyl conjugates was achieved using a convergent approach whereby the endoperoxide and tetrazole or saccharyl building blocks were separately prepared and then linked to form the desired endoperoxide-based targets. In our library, the chemical diversity is introduced in the cyclohexyl moiety (Fig. 1). Synthetic trioxolanes (compounds LC28, LC50, LC67, LC68, LC92, LC93, LC94, LC95, LC129, LC130, LC131, LC132, LC136, LC142, MIS13, MIS14, MIS15, MIS16; Fig. 1: Scheme 1) and tetraoxanes (compounds LC140, LC163; Fig. 1: Scheme 2) were prepared using the synthetic approaches depicted in Schemes 1 and 2.

### Cytotoxicity against mammalian cells

In order to evaluate the selectivity of compounds for parasite over mammalian cells, their cytotoxicity was assessed in human hepatocellular carcinoma (HepG2) and hamster lung (V79) cells. All tested compounds presented low or undetectable cytotoxicity in both cell lines. The selectivity index (SI) for each compound is expressed by the ratio between its cytotoxicity and antiplasmodial activity (SI = LD_50_ in mammalian cells/IC_50_ *P. falciparum*). At a maximum dose of 1 mM, only MIS13 and MIS14 revealed some in vitro cytotoxicity, inducing a decrease in HepG2 survival of ≅ 30%. The remaining compounds displayed high selectivity indexes (SI > 273) (Additional file 1).

### In vitro activity of new endoperoxides

Initially, all 21 synthesized endoperoxides were screened for their in vitro anti-malarial activity against chloroquine-susceptible (3D7) and multidrug-resistant (Dd2) *P. falciparum* strains (Table 1; Additional file 1), using a whole-cell SYBR Green I based assay. The IC_50_ values were calculated from at least 3 experiments, carried out in triplicates. The obtained IC_50_ for the control drug CQ against 3D7 and Dd2, fell within the expected range (Table 1). Among the 21 compounds, 12 showed sub-micromolar activity (IC_50_ < 1 μM) against both 3D7 and Dd2 strains, with 9 exhibiting IC_50_ values < 100 nM (LC92, LC129, LC130, LC131, LC132, LC136, LC163, MIS13 and MIS14) (Additional file 1). These 9 compounds were further tested against two ART-R strains (IPC5202 and IPC4912) and, except for MIS14, presented very promising anti-malarial activity, with IC_50_ values ranging from 0.3 to 71.1 nM (Table 1). In addition to sub-micromolar activity, these eight compounds also demonstrated high selectivity towards the parasite (Additional file 1) and a low resistance index (RI; ranging from 0.10 to 2.43), indicating absence of cross resistance with ARTs (Table 1). Thus, compounds LC92, LC129, LC130, LC131, LC132, LC136, LC163 and MIS13 were selected for further studies.

**Table 1:**
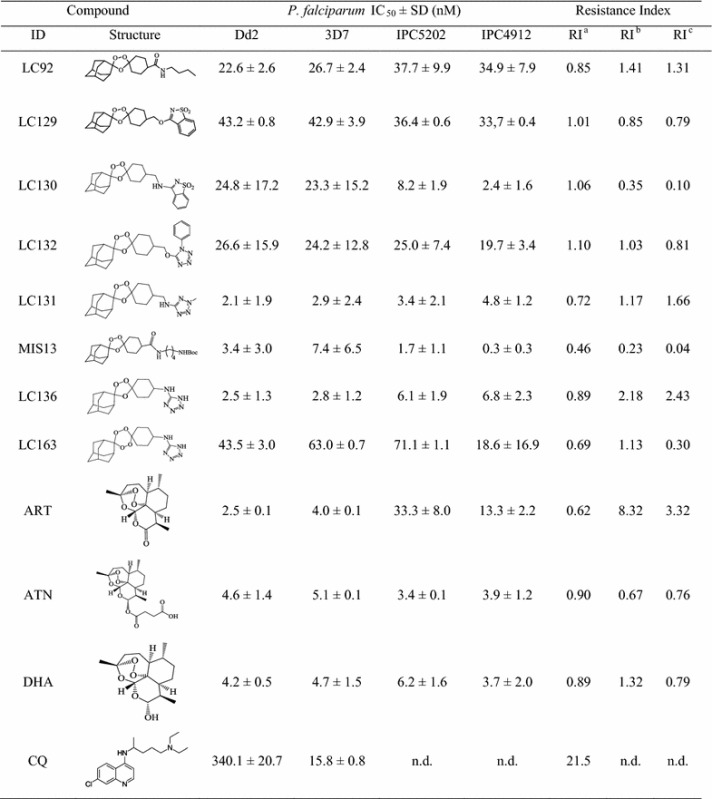
Antiplasmodial activity in vitro (IC50) against resistant and sensitive *P. falciparum* strains

*SD* standard deviation, *ART* artemisinin, *ATN* artesunate, *DHA* dihydroartemisinin, *CQ* chloroquine, *n.d.* not determined

RI^a^: resistance index = IC_50_ (Dd2)/IC_50_ (3D7)

RI^b^: resistance index = IC_50_ (IPC5202)/IC_50_ (3D7)

RI^c^: resistance index = IC_50_ (IPC4912)/IC_50_ (3D7)

### Ring and mature-stage survival rate

**Fig. 2:**
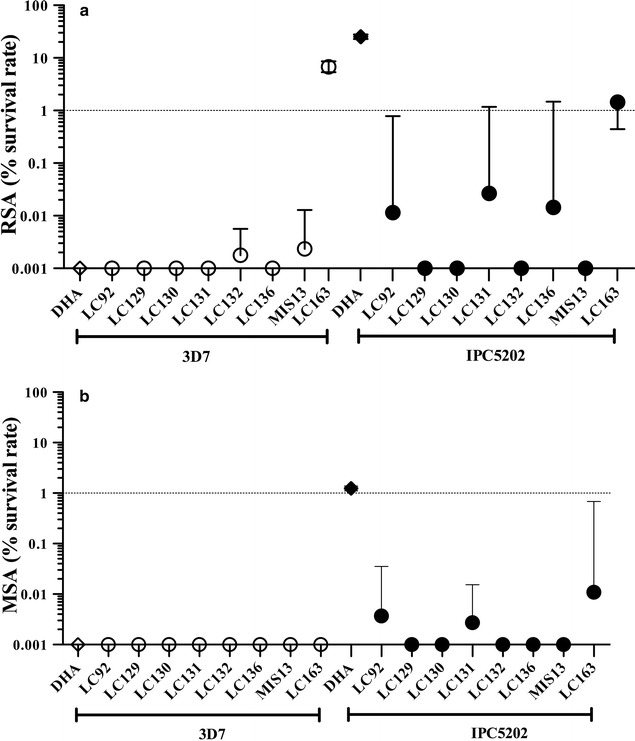
Ring-stage (RSA) and mature-stage (MSA) survival rate, expressed as the percentage of viable parasites. Rings (**a**) and trophozoites (**b**) were treated with a pulse of 700 nM of DHA or (10 × IC_50_) synthetic endoperoxides in strains 3D7 and IPC5202

Resistance to ARTs in vivo is characterized by a delayed parasite-clearance time. Up to now this phenotype could not be evidenced using the standard in vitro 48–72 h inhibitory assay, expressed as IC_50_ [13, 21, 27–31], probably due to the elevated resistance of the very young-ring stages of the parasites. Hence, the phenotypic assay, ring-stage survival assay (RSA), was used to assess the activity of the 8 compounds selected (LC92, LC129, LC130, LC131, LC132, LC136, LC163 and MIS13). Young ring-stage survival of *P. falciparum* parasites carrying the K13 R539T polymorphism (IPC5202) associated with high RSA and 3D7 were challenged during 6 h with 700 nM DHA or a concentration of 10xIC50 of each of the tested compounds. Generally, the 8 endoperoxides demonstrated higher activity than DHA against the resistant strain (Fig. 2a). It is generally accepted that a survival rate RSA < 1% corresponds to a susceptible behaviour. The 8 endoperoxides were able to reduced RSA < 1% (Fig. 2a) in both ART-R and ART-S strains (IPC5202 and 3D7). On the other hand, DHA exposure resulted in a higher survival rate of the ART-R (up to 25%) than the susceptible 3D7. Concerning the new trioxolanes, no cross-resistance with DHA was apparent for parasites carrying the K13 R539T mutation (Table 1). However, for the tetraoxane LC163 both ART-S and ART-R strains exhibited ring-stage survival above 1% (6.9 ± 1.6 and 2.0 ± 1.9, respectively), although no cross-resistance was observed (Table 1). Mature-stages of both the ART-S and ART-R parasites exhibited full susceptibility to all of the 8 selected endoperoxides, which appear to offer a better performance than DHA (Fig. 2b).

### In vivo anti-malarial activity

The 8 best performing compounds were administered orally at 50 mg/kg/day and demonstrated a high inhibition capacity, with parasitaemia ranging from 0 to 0.19 ± 0.12% on day 5 post-infection (Table 2). The efficacy difference was very significant (P < 0.0002), compared to the vehicle-treated mice; the control inhibited parasitaemia by 1.51% ± 0.22 (Table 2). On day 10 post-infection, mice in the control group developed significant parasitaemia (5.65–6.6%), whereas 5 of the compounds led to an appreciable reduction in parasitaemia (0.31 ± 0.10–1.32 ± 1.24%) and the remaining 3 compounds completely suppressed parasitaemia (Table 2). Although at a low level, in the group treated with LC92 5 animals developed parasitaemia from day 5 onwards. In the group treated with MIS13 only 1/5 mice presented parasitaemia on day 5, and by day 10 the five mice were cured. Even though MIS13 was the most cytotoxic in vitro against HepG2 cells, from the range of 21 compounds tested, no adverse effects were observed in vivo. During the experimental procedure, the animals were monitored daily and no adverse effects were observed for any of the tested compounds. Appreciable in vivo anti-malarial activity was observed for the two sacharyl substituted compounds (LC129, LC130). LC129 kept parasitaemia at a low level, but failed to completely suppress it. On the other hand, LC130 was able to suppress parasitaemia until day 5 but recrudescence occurred on day 7 (2/5 mice were parasitemic) and by day 10 all animals presented parasitaemia (Table 2), although significantly lower than in the control group (P = 0.017). In the group treated with LC132 and LC163 recrudescence also occurred on day 10. However, a strong in vivo anti-malarial effect against *P. berghei* was observed with LC131 and LC136, with complete suppression of parasitaemia throughout the experiment (10 days), suggesting that sufficient plasma concentration was attained, hence indicating a promising bioavailability and pharmacokinetic profile.

**Table 2:** In vivo anti-malarial activity against *Plasmodium berghei*

Compound	Dose (mg/kg/day)	Mean parasitaemia ± SD (% inhibition of parasite growth)^b^		
		Day 5	Day 7	Day 10
Control^a^		1.51 ± 0.22	3.58 ± 0.81	5.65 ± 0.43
LC92	50	0.02 ± 0.05 (98.34)	0.46 ± 0.60 (86.99)	0.77 ± 0.26 (86.27)
LC129	50	0.19 ± 0.12 (87.60)	1.76 ± 1.36 (50.69)	1.32 ± 1.24 (76.67)
LC130	50	0 (100)	1.4 ± 0.94 (60.83)	1.32 ± 0.24 (76.67)
LC131	50	0 (100)	0 (100)	0 (100)
LC132	50	0 (100)	0 (100)	0.31 ± 0.10 (94.46)
LC136	50	0 (100)	0 (100)	0 (100)
MIS13	50	0.11 ± 0.19 (92.56)	0.05 ± 0.10 (98.60)	0 (100)
LC163	50	0 (100)	0 (100)	0.2 ± 0.13 (96.46)

*SD* standard deviation

^a^Control: treated with PBS 1% DMSO

^b^Parasitaemia reduction compared to untreated control group

## Discussion

The new peroxide-type compounds tested have shown low or undetectable cytotoxicity against human hepatocellular carcinoma (HepG2) and hamster lung (V79) cell lines. It is generally accepted that, if SI > 10, the observed pharmacological activity is not due to cytotoxicity [32, 33]. Since the SI values calculated for the compounds are considerably higher, that the activity exhibited by the compounds is unlikely due to general cellular toxicity, but rather due to specific antiplasmodial activity. The eight endoperoxides selected from our library that met the criteria—SI > 100 and IC_50_ < 100 nM [34] were evaluated for in vitro and in vivo efficacy, demonstrating IC_50_ values in the range of those exhibited by ARTs and a strong curative effect in vivo, with a parasitaemia reduction on day 10 above 76% (Tables 1, 2). These 8 compounds were particularly active against intra erythrocytic stages, across all four *P. falciparum* strains tested, with IC_50_ values ranging from 0.3 to 71.1 nM and high selectivity for the parasite. Even though LC136 presented RIs > 1 (up to 2.43), the values fall within the range of those exhibited by ARTs for the same parasite strains (0.62–8.32; Table 1).

Results show that the nature of the cyclohexyl substituent affects antiplasmodial activity. For instance, LC92 exhibited IC_50_ values up to 11 times higher than the control drugs (ART derivatives) while its counterpart MIS13 was shown to be as effective as ARTs across the four parasite strains, with no apparent cross resistance. This increased activity probably arises from an improvement in pharmacokinetic properties due to: (i) a more substituted amino functionality that increases the overall hydrophilicity and favours protonation in acidic environments; and (ii) to the BOC-protection of the side chain that facilitates the transport of MIS13 through the cell membrane (compared to LC92) [35]. Although MIS13 induced a slight cytotoxicity in vitro (30% reduction in survival of HepG2 cells at 1 mM), no adverse effects were observed in vivo. Only 1/5 animals treated with MIS13 presented parasitaemia on day 5, which gradually decreased until 0% by day 10, whereas in the group treated with LC92 all animals presented residual parasitaemia during the 10 days.

Compounds LC131, LC132 and LC136, comprising a tetrazole moiety linked to the trioxolane pharmacophore, exhibited an excellent anti-malarial profile, both in vitro and in vivo. Tetrazole-containing drugs and drug-candidates are thriving in medicinal chemistry [36, 37] and many are effective therapeutic agents for various pathogenesis, showing a wide range of pharmacological activities, such as antimicrobial, antibacterial, antifungal, and antitubercular [38–41]. The tetrazole moiety has been described as metabolically stable, featuring in the structure of several drugs and drug-candidates, usually as a surrogate for the carboxylic acid functionality, and generally improving the pharmacokinetic profile [42, 43]. The higher metabolic stability conferred by the tetrazole [42–46] could be improving the pharmacological properties or increasing accumulation (due to the electron-withdrawing properties). In line with this, a better in vivo anti-malarial profile for the tetrazole conjugates LC131, LC132, LC136 and LC163, was observed, particularly for trioxolanes LC131 and LC136, which exhibited a curative effect in mice with total suppression of parasitaemia from day 5 to day 10. To assess the nature of the tetrazole moiety contribution to the anti-malarial activity, the tetrazole building blocks (LC133 and LC126II; Additional file 1) were separately tested and revealed to be devoid of antiplasmodial activity. The ether-linked tetrazolyl–trioxolane conjugate LC132 was up to 12 times less active than LC131 or LC136 and, in vivo, allowed recrudescence on day 10. Probably, this compound is less stable due to the possibility of thermally-induced 1,3-isomerization of the alkyl-trioxolane moiety [47], as the C(alkyl)-*O* bond is relatively weak due to the electron withdrawing effect of the tetrazole heterocycle. Comparatively, the amino-linked conjugates LC131 and LC136 are chemically more stable. LC131 and LC136 exhibited an exceptionally good in vitro anti-malarial profile against both ART-R and ART-S parasites, comparable to that of the control drugs ART, ATN and DHA. The ability of the amino group to protonate in the acidic environment of the food vacuole probably confers additional anti-malarial efficacy by facilitating accumulation, placing the two new compounds as promising drug candidates. In conjugates LC131 and LC136 the heteroaromatic substituent is a 2-substituted 5-aminotetrazole (in LC136 the tetrazole ring exhibits tautomerism, with the 2-*H* tautomer being favoured in the gas phase) [48]. An additional interest of this substitution pattern resides in the potential of 2,5-disubstituted tetrazoles to act as probes. It was demonstrated that, upon narrow-band UV-induced irradiation, 2,5-dissubstituted tetrazoles generate nitrile imine dipolar species in situ, via extrusion of N_2_, which may then react with an alkene dipolarophile through a 1,3-dipolar cycloaddition reaction [49, 50]. Thus, the trioxolane–tetrazole conjugates LC131 and LC136 could also be developed as tags for mechanistic studies.

Compound LC163 is a tetraoxane–tetrazole conjugate structurally related to the trioxolane LC136. The results show that replacement of the trioxolane by a tetraoxane ring, as the pharmacophore, leads to a decreased anti-malarial activity, both in vitro and in vivo. Even though the RIs for LC163 are low, the corresponding IC_50_ values for the resistant strains IPC4912 and IPC5202 are, respectively, 3 and 42 times higher than those found for trioxolane LC136. The reason for this difference is still elusive [51], so detailed structural studies on both compounds are planned.

The saccharyl substituent present in compounds LC129 and LC130 is also electron withdrawing and metabolically stable. Additionally, the saccharyl system is thermally and photochemically more stable than the tetrazole system and is generally found to improve the overall stability of the molecule [27], although it is worth noting that LC129 is prone to Chapman-type isomerization [27]. The anti-malarial profile exhibited by the trioxolane–saccharyl conjugates LC129 and LC130 was slightly inferior to that of their tetrazole analogues (Tables 1, 2). As for trioxolane–tetrazoles, the saccharyl building block was tested separately and revealed to be devoid of antiplasmodial activity (Additional file 1). Hence, the anti-malarial activity demonstrated by the trioxolane–saccharyl conjugates can be ascribed to the presence of the trioxolane pharmacophore.

Structural analogies between DHA, trioxolanes and tetraoxanes may accommodate similar modes of action, hence some level of cross resistance [10, 28–31, 52]. In the light of this, it was investigated whether parasites expressing variant forms R539T (IPC502) and I543T (IPC4912) of K13 are cross-resistant to the newly synthesized trioxolanes and tetraoxanes.

ARTs resistance cannot be evidenced by the standard in vitro IC_50_ assay [13, 21, 27–31]. Critical for the development of anti-malarials is the evaluation of their activity against ART-R parasites, which has been defined as delayed parasite clearance in patients [53]. Slow parasite clearance of *P. falciparum* malaria in patients results from reduced ring-stage susceptibility [53–56], this increasing the need for new compounds with a low RSA. The mutation R539T (present in IPC5202) is one of the k13 mutations that confer high levels of DHA resistance in vitro [31, 52, 57]. We explored the susceptibility of the IPC5202 ring and mature-stage parasites (RSA and MSA) to the best performing compounds. As depicted in Fig. 2, the endoperoxides selected demonstrated higher activity than DHA, both against ring and mature stages. As expected, DHA exposure resulted in a higher survival rate of IPC5202 (up to 25%) than for 3D7 [58, 59], though lower than in other reports [60]. A survival rate RSA < 1% is generally considered as susceptible behaviour. The tested trioxolanes were able to reduce ring survival to less than 1% in both resistant and susceptible strains (IPC5202 and 3D7), hence no cross-resistance with DHA is apparent, even in parasites carrying the K13 mutation R539T. This notable observation indicates that our compounds have an improved range of activity, compared to other trioxolanes in use, namely the registered drug OZ277, which is compromised by K13 mutations [31]. Regarding the tetraoxane LC163, both the K13 wild type (3D7) and mutant (IPC5202) parasites presented RSA > 1% (though not significantly different). These observations are in agreement with data recently published showing that a tetraoxane also allowed growth above 1% in RSA assay in a strain carrying K13 R538T mutation [61]. The results indicate therefore that the anti-malarial activity of the newly synthesized endoperoxides is not compromised by the K13 mutation R539T, a mutation that confers high levels of in vitro resistance and has been associated with delayed parasite clearance in patients [8, 62–65]. The mature-stage assay (MSA) evidenced nearly full susceptibility of the two strains to all of the eight compounds (Fig. 2). Thus, all compounds performed better than DHA. Typically, DHA allows a ≅ 1% of viable mature-stage [13, 21]. ARTs resistance phenotypes are associated with a decreased susceptibility of the ring stage to enter dormancy, a decreased sensitivity of mature-stage parasites and a faster recovery from dormancy [66, 67]. Currently these parameters are being addressed in order to gather more information regarding the mode of action of the more promising compounds.

As expected, no cross-resistance of the proposed compounds with quinolone-type anti-malarials is foreseeable, as the calculated RIs for Dd2 and 3D7 were ≅1 (Table 1), which is considerably lower than the RI determined for CQ (21.5) [20, 68].

## Conclusion

The compounds LC131, LC136 and MIS13 demonstrated to be the most promising; they showed nanomolar activity against ART-resistant *P. falciparum* parasites, negligible toxicity towards mammalian cells, totally suppressed *P. berghei* parasitaemia in mice and showed no cross resistance with CQ and ARTs.

These compounds can be easily prepared from relatively cheap starting materials. For instance, LC136 requires only 3 synthetic steps. On the other hand, ART-derivatives are obtained from the expensive natural product artemisinin through semi-synthesis. Thus, besides the excellent pharmacologic properties, an anti-malarial peroxide-type formulation based on trioxolane LC136 could be cheaper than those based on current ART-derivatives, while offering a comparable, or even better, anti-malarial profile.

## Additional file

**Additional file 1.** Synthetic procedures and experimental details for the preparation and chemical characterization of compounds.
