# Supplementary material for: New endoperoxides highly active in vivo and in vitro against artemisinin-resistant Plasmodium falciparum
Source: Malar J. 2018 Apr 3;17:145. doi: 10.1186/s12936-018-2281-x (PMC5883364; doi:10.1186/s12936-018-2281-x)
Supplement: Supplementary file 1 — Additional file 1. Synthetic procedures and experimental details for the preparation and chemical characterization of compounds. [file 12936_2018_2281_MOESM1_ESM.docx]

**Additional file**

**Title: New endoperoxides highly active *in vivo* and *in vitro* against *Plasmodium falciparum* artemisinin-resistant parasites**

Lis Lobo^1,5,#^, Lília I. L. Cabral^2,3#^, Maria Inês Sena^2^, Bruno Guerreiro^2,3^, António Sebastião Rodrigues^4^, Valter Ferreira de Andrade-Neto^5^, Maria L. S. Cristiano^2,3,*^, Fátima Nogueira^1,*^

^1^Global Health and Tropical Medicine, GHTM, Unidade de Ensino e Investigação de Parasitologia Médica, Instituto de Higiene e Medicina Tropical, IHMT, Universidade Nova de Lisboa, UNL, Rua da Junqueira nº 100, 1349-008 Lisboa, Portugal;

^2^Centre of Marine Sciences, CCMAR, Universidade do Algarve, UAlg, Campus de Gambelas, 8005-139 Faro, Portugal;

^3^Departmento de Química e Farmácia, Faculdade de Ciências e Tecnologia, FCT, Universidade do Algarve, UAlg, Campus de Gambelas, 8005-139 Faro, Portugal;

^4^Centre for Toxicogenomics and Human Health, Genetics, Oncology and Human Toxicology, Nova Medical School/Faculdade de Ciências Médicas, Universidade Nova de Lisboa, Rua Câmara Pestana, nº 6 Edifício CEDOC II, Room 2.24 1150-008 Lisboa, Portugal;

^5^Laboratório de Biologia da Malária e Toxoplasmose, Departamento de Microbiologia e Parasitologia, Universidade Federal do Rio Grande do Norte, Natal, Rio Grande do Norte, Brasil.

^#^Both authors contributed equally to the work

*Corresponding authors:

[fnogueira@ihmt.unl.pt](mailto:fnogueira@ihmt.unl.pt)

Global Health and Tropical Medicine, GHTM, Unidade de Ensino e Investigação de Parasitologia Médica, Instituto de Higiene e Medicina Tropical, IHMT, Universidade Nova de Lisboa, UNL, Rua da Junqueira nº 100, 1349-008 Lisboa, Portugal;

Telephone +351 213652600

Fax +351 213632105

[mcristi@ualg.pt](mailto:mcristi@ualg.pt)

Centre of Marine Sciences, CCMAR, Universidade do Algarve, UAlg, Campus de Gambelas, 8005-139 Faro, Portugal

***S.1. Synthetic procedures and experimental details for the preparation and chemical characterization of compounds.***

***S.1.1. General methods and analytical techniques***

Commercial reagents were used as purchased. When required, solvents were dried following standard procedures. ^1^H and ^13^C-NMR spectra were recorded on a 400 MHz NMR spectrometer. ^1^H-NMR-chemical shifts are referred to the residual signal of CDCl_3_ (δH 7.27); ^13^C-NMR- chemical shifts are referred to the CDCl_3_ signal (δC 77.0), or to TMS, as internal standard. Thin-layer chromatography was carried out on silica gel 60 F254 plates (AL TLC 20x20). Column chromatography was performed on Silica Gel 60 (0.04 – 0.063 mm). IR spectra were recorded on a Tensor 27 FT/IR spectrometer in the 600–3800 cm^-1^ range. Melting points (°C) were obtained on a “SMP3 Melting Point Apparattus, and are uncorrected.

***S.1.2. Preparation of intermediate building blocks***

The synthetic approaches followed to the preparation of intermediate blocks, used for the synthesis of final target compounds, are illustrated in Schemes S1-S3. Synthetic procedures for each compound prepared are also provided in this section.

***S.1.2.1. Preparation of 3-chloro-1,2-benzisothiazole-1,1-dioxide, LC60.***

**Scheme S1**: Reagents and conditions: i) PCl_5_, 180 ºC.

The experimental procedure used has been reported previously (Araújo et al., 2002). From saccharin (56 mmol) and phosphorus pentachloride (66 mmol), heated at 200 ºC. Colourless needles from ethanol (63% yield); m.p. 143-145 ºC. IR *ν_max_* (cm^−1^): 1724, 1654, 1603 (C=C), 1346 (SO_2_), 775 (Ar-H) and 692 (C-Cl); ^1^H-NMR (400 MHz, CDCl_3_): δ 7.85 (4H, m, Ar-H) ppm. Found: C, 41.5%; H, 2.0%; N, 6.9%; calcd for C_7_H_4_NO_2_SCl: C, 41.7%; H, 2.0%; N, 7.0%. MS (EI), *m*/*z* 201 [M]^+^.

***S.1.2.2. Preparation of 1-phenyl-1H-tetrazole-5-one (LC133), 1-methyl-1H-tetrazole-5-amine (LC126I), and 2-methyl-2H-tetrazole-5-amine (LC126II)***

**Scheme S2**: Reagents and conditions: i) NaOH (5M), r.t.; ii) Dimethylsulfate, NaOH/H_2_O, phenolphthalein, 100ºC.

***1-phenyl-1H-tetrazol-5-one, LC133***

5-Chloro-1-phenyl-tetrazole (1 eq) was added to a solution of sodium hydroxide (5M, 10 mL). The reaction mixture was stirred at room temperature for 24 h. The resulting solution was cooled to room temperature and acidified by addition of HCl (aq) (10%; pH≈1). A precipitate was formed, filtered and washed with chloroform and hexane to give the product (90% yield) as a colourless powder; m.p. 97-99 ºC; ^1^H-NMR (400 MHz, CDCl_3_): δ 7.57 (m, 2H), 7.66 (s, 1H), 7.70 (d, 1H) ppm; MS (IE), *m/z*: 162,05 [M]^+^.

***1-methyl-1H-tetrazole-5-amine, LC126I***

A solution of sodium hydroxide (20%) was added dropwise to a suspension of 5-aminotetrazole monohydrate (120 mmol) in water (30 mL), with a drop of phenolphthalein. The mixture was stirred until complete dissolution of the suspended material. Dimethyl sulphate (110 mmol) was then added in small portions, keeping an alkaline medium through addition of aqueous sodium hydroxide. The final mixture was refluxed for 1 h, cooled, and finally left in an ice bath for 48h. The colourless needles formed were filtered and dried to afford the desired compound (51% yield); m.p. 220-221 ºC. ^1^H-NMR (400 MHz, CDCl_3_): δ 4.15 (3H, s) ppm; MS (EI), *m/z* 99 [M]^+^.

***2-methyl-2H-tetrazole-5-amine, LC126II***

The filtrate from 1-methyl-1*H-*tetrazole-5-amine **LC126I** synthesis was evaporated under reduced pressure to afford a solid residue. Water (50 mL) was added, and the mixture was then extracted with diethyl ether (3 x 50 mL). The organic extract was dried over anhydrous sodium sulphate, filtered, and the filtrate evaporated to afford colourless crystals. Recrystallization from diethyl ether gave the desired compound as colourless needles (25% yield); m.p. 104.5-105.5 ºC. ^1^H-NMR (400 MHz, CDCl_3_): δ 3.32 (3H, s) ppm; MS (EI), *m/z* 99 [M]^+^.

***S.1.2.3. Preparation of tert-butyl(4-aminobutyl)carbamate***

**Scheme S3**: Reagents and conditions: i) Boc_2_O, 1,4-dioxane, r.t.

A solution of di-tert-butyl dicarbonate (2.50 x 10^-2^ mol) in 1,4-dioxane (100 mL), under stirring, was added by cannula, over 3 hours, to a stirring solution of 1,4-diaminobutane (1.40 x 10^-1^ mol) in 1,4-dioxane (100 mL). The final reaction mixture was stirred at room temperature for 20 h and then concentrated under reduced pressure. Water was added, and the conjugate formed precipitated. The aqueous residue was extracted with DCM (2 x 30 mL). The combined organic extracts were dried over anhydrous MgSO_4_, filtered, and the filtrate was evaporated to dryness under reduced pressure to give a clear oil (97 % yield). ^1^H-NMR (400 MHz, CDCl_3_): δ 1.42 (s, 9H), 1.48 (d, 4H), 2.71 (s, 2H), 3.10 (s, 2H) ppm; MS (MALDI-TOF), *m/z* 189,17 [M+H]^+^.

***S.1.3. Preparation of trioxolanes***

The synthetic approach followed to trioxolanes is illustrated in Schemes S4-S8. Synthetic procedures for the preparation of each compound are also provided in this section.

**Scheme S4**: Reagents and conditions: i) Pyridine, MeONH_2_, MeOH, r.t; ii) 1,4-Cyclohexane, O_3_, DCM/Pentane, -78 ºC; iii) Ethyl 4-oxocyclohexanecarboxylate, O_3_, DCM/Pentane, -78 ºC.

***O-methyl-2-adamantanone oxime, LC29***.

To a solution of 2-adamantanone (30 mmol) in methanol (30 mL) were added pyridine (55.6 mmol) and methoxylamine hydrochloride (45.0 mmol). The reaction mixture was stirred at room temperature for 48 h. The final mixture was concentrated and then diluted with DCM (50 mL) and water (50 mL). The organic layer was separated and the aqueous layer was extracted with DCM (30 mL). The combined organic extracts were washed with aqueous HCl (1 M; 30 mL x2), then with saturated aqueous NaCl (30 mL). The final organic extract was dried over MgSO_4_, filtered and concentrated under reduced pressure to give *O*-methyl-2-adamantanone oxime (89% yield) as a colourless solid (m.p. 69-70 ºC). ^1^H-NMR (400 MHz, CDCl_3_): δ 1.78-1.97 (12H, m), 2.53 (1H, s), 3.45 (1H, s), 3.81 (3H, s) ppm; MS (MALDI-TOF), *m/z* 180.02 [M]^+^.

***S.1.3.1. Synthesis of 1,2,4-trioxolanes***

***General procedure 1: Preparation of Adamantyl-1,2,4-trioxolanes LC50 and LC67.*** Trioxolanes were prepared by coupling O-methyl-2-adamantanone oxime (2) with a cyclohexanone derivative, through ozonolysis.

Ozone, produced with an ozone generator Sander Labor-Ozonizator 301.7 (0.5 L/min O_2_, 140 V), was passed through a solution of dichloromethane at –78 ºC and flushed into a solution of *O*-methyl ketone oxime and a ketone, in pentane/dichloromethane (6:4), at 0 ºC. After completion, the solution was flushed with nitrogen for 5 min and concentrated under reduced pressure, at room temperature, to give a crude material that was purified by column chromatography.

***Adamantyl-1,2,4-trioxolane, LC50***.

A solution of *O*-methyl 2-adamantanone oxime (8.4 mmol) and 1,4-cyclohexanedione (11 mmol) in pentane (60 mL) and dichloromethane (40 mL) was treated with ozone, as described in general procedure 1. The crude product was purified by column chromatography (silica gel; ethyl acetate/n-hexane 1/9) to give product **LC50** (42% yield) as a colourless solid; m.p. 127-128 ºC; ^1^H-NMR (400 MHz, CDCl_3_): δ 1.69-2.02 (m, 14H), 2.14 (t, 4H), 2.51 (t, 4H) ppm; ^13^C-NMR (100 MHz, CDCl_3_): 25.9, 26.31, 31.09, 32.59, 34.25, 35.70, 36.18, 37.35, 106.46, 111.95, 208.90 ppm; MS (EI), *m/z* 278.9 [M]^+^.

***Adamantyl-1,2,4-trioxolane, LC67***.

A solution of *O*-methyl 2-adamantanone oxime (20 mmol) and ethyl 4-oxocyclohexanecarboxylate (20 mmol), in pentane (60 mL) and DCM (40 mL), was treated with ozone, as described in general procedure 1. The crude product was purified by column chromatography (silica gel, ethyl acetate/n-hexane 1/9) to afford trioxolane **LC67** as a colourless oil (46% yield). ^1^H-NMR (400 MHz, CDCl_3_): δ 1.26 (3H, t), 1.70-1.76 (11H, m), 1.92-2.03 (12H, m), 2.33 (1H, m), 4.15 (2H, dd) ppm; MS (MALDI-TOF), *m/z* 337.34 [M]^+^.

***S.1.3.2. Preparation of trioxolanes LC68, LC93, LC94, LC95, LC129, LC130, LC131, LC132, MIS13, MIS15, LC92, LC142***

**Scheme S5**: Reagents and conditions: i) LiBH_4_, Et_2_O, LiBH(Et)_3_, r.t.; ii) Phthalimide, Ph_3_P, DIAD, THF, 0 ºC; iii) Hydrazine hydrate, Chloroform/MeOH, 60 ºC; iv) **LC60**, THF, 60 ºC; v) LC60, TEA, Toluene, 45 ºC; vi) Triethyamine, mesyl chloride, THF, 60 ºC; vii) 5-Chloro-1-phenyl-tetrazole, TEA, Toluene, 45 ºC; viii) MeOH, KOH (3M), 60 ºC; ix) LC64, EDC, HOBt, N-methylmorpholine, DCM, r.t.; x) Trichloroacetic acid, DCM, H_2_O, r.t.; xi) Butylamine, EDC, HOBt, N-Methylmorpholine, DCM, r.t.; xii) 5-Aminotetrazole monohydrate, EDC, HOBt, N-Methylmorpholine, DCM, r.t.

***Adamantyl-1,2,4-trioxolane LC93***.

A solution of **LC67** (11.3 mmol), lithium borohydride (11.3 mmol, 2M in THF) and lithium triethylborohydride (1.13 mmol, 1M in THF) in ether (15 mL) was stirred overnight, at room temperature. The reaction mixture was diluted with ether (5 mL), washed with aqueous NaOH (3M; 2 x 10 mL), then with brine and water (2 x 10 mL). The organic extract was dried over MgSO_4_, filtered, and concentrated under reduced pressure to give product **LC93** (90% yield) as a yellow crystalline solid; m.p. 99-101 ºC. ^1^H-NMR (400 MHz, CDCl_3_): δ 1.25 (2H, m), 1.51-2.08 (21H, m), 3.46 (2H, t) ppm; MS (MALDI-TOF), *m/z* 318.30 [M+Na]^+^.

***Adamantyl-1,2,4-trioxolane LC94***.

A solution of **LC93** (2.8 g, 9.52 mmol) in dry THF (25 mL) was cooled to 0 °C. Ph_3_P (3.5 g, 1.33 mmol), phthalimide (1.55 g, 10.5 mmol) and DIAD (2.6 mL, 1.33 mmol) were gradually added. The mixture was stirred at room temperature for 24 hours. The solvent was then evaporated to dryness and the crude product was purified by column chromatography (silica gel, ethyl acetate/n-hexane 1/9) to give product **LC94** (80% yield) as a white powder (m.p. 149-151 ºC). ^1^H NMR (300 MHz, CDCl_3_): δ 1.30-1.34 (2H, m), 1.51-2.08 (21H, m), 3.55 (2H, d), 7.71 (2H, m), 7.84 (2H, m) ppm; MS (MALDI-TOF), *m/z* 462.19 [M+K]^+^.

***Adamantyl-1,2,4-trioxolane LC95***.

A solution of **LC94** (3.20 g, 7.56 mmol) and hydrazine monohydrate (1.45 g, 45.4 mmol) in chloroform and methanol (7:3, 50 mL total) was heated at 60 ºC for 35 h. The reaction mixture was cooled to room temperature and filtered to remove solid by-products. The filtrate was washed with water (50 mL) and brine (50 mL), dried over MgSO_4_, filtered, and concentrated to give product **LC95** (77% yield) as light yellow oil. ^1^H NMR (400 MHz, CDCl_3_): δ 1.14-1.33 (3H, m), 1.68-1.96 (22H, m), 2.54 (2H, d) ppm; MS (MALDI-TOF), *m/z* 293.20 [M]^+^.

***Adamantyl-1,2,4-trioxolane LC130***.

Compound **LC60** (0.71 g, 3.53 mmol) was added to solution of LC95 (1 g, 3.41 mmol) in dry THF (20 mL). The solution was stirred at 60 ºC until all of the starting material had disappeared. The reaction mixture was cooled to r.t. and evaporated. Recrystallization from ethanol gave the desired compound as yellow crystalline solid (61% yield); m.p. 152-154 ºC. ^1^H RMN (CDCl_3_): δ 1.21-1.30 (2H, m), 1.65-2.0 (22H, m), 3.43 (2H, s), 7.68 (1H, d), 7.75 (2H, dd), 7.89 (1H, d) ppm; MS (MALDI-TOF), *m/z* 481.16 [M + Na]^+^.

***Adamantyl-1,2,4-trioxolane LC132***

To a solution of **LC93** (1.83 mmol) in THF (10 mL) was added mesyl chloride (2.0 mmol) and triethylamine (3.65 mmol). The solution was stirred at room temperature for 3 hours. Then a solution of **5-chloro-1-phenyl-tetrazole** (2.75 mmol) in THF (10 mL) was added dropwise to the stirred suspension, over 30 minutes. The mixture was stirred at 65 ºC for 24 hours. Excess solvent was then removed. Recrystallization from ethanol gave **LC132** as a white solid (32% yield). m.p. 82-84 ºC. ^1^H-NMR (400 MHz, CDCl_3_) 7.73 (d, 2H), 7.57 (t, 2H), 7.48 (t, 1H), 4.50 (d, 2H), 2.01 (d, 7H), 1.71-1.86 (m, 13H), 1.39-1.46 (m, 2H), 1.27 (s, 1H) ppm; MS (MALDI-TOF), *m/z* 477.11 [M+K]^+^.

***Adamantyl-1,2,4-trioxolane LC129***.

Compound **LC60** (4.08 mmol) was added to a solution of compound **LC93** (3.4 mmol) in dry toluene (30 mL). The solution was stirred at 45 ºC for 15 minutes, followed by addition of triethylamine (6.8 mmol) until disappearance of all of the starting material. The precipitate of triethylamine hydrochloride was filtered off and the filtrate was evaporated to give a yellow crystalline solid, which was recrystallized from ethanol (50% yield); m.p. 150-151 ºC. ^1^H-NMR (400 MHz, CDCl_3_): δ 1.59- 1.44 (m, 2H), 2.05- 1.71 (m, 21H), 4.45 (d, 2H), 7.74 (d, 1H), 7.78 (d, 2H), 7.91 (d, 1H) ppm; MS (EI), *m/z* 482 [M + Na]^+^.

***Adamantyl-1,2,4-trioxolane LC68***.

To a solution of **LC67** (4 mmol) in methanol (15 mL) was added a solution of potassium hydroxide (20 mmol) in water (6 mL). The mixture was refluxed for 6 hours. Then, the solution was allowed to cool to room temperature and was concentrated under reduced pressure. The crude was dissolved in water (50 ml) and then washed with dichloromethane (30 ml). The aqueous layer was acidified to pH 1 with concentrated hydrochloric acid and then extracted with dichloromethane (3 x 40 ml). The combined organic phases were washed with brine (30 ml), dried over Na_2_SO_4_, filtered, and concentrated under reduced pressure to give the pure compound as a white solid (95% yield). m.p. 158-159 °C. ^1^H RMN (CDCl_3_): δ 1.19-1.26 (3H, m), 1.61-1.94 (19H, m), 2.30 (1H, m) ppm; MS (EI), *m/z* 306.85 [M]^-^.

***General Procedure 2: Preparation of adamantyl-1,2,4-trioxolanes MIS13, LC92 and LC142*.** EDC.HCl (1.5eq), HOBt (1.5eq) and NMM (2.1eq) were added to **LC68** (1eq) in DCM (15ml) at 0ºC. The solution was stirred at room temperature for 3hrs, under N_2_, before different amines (1.5eq) were added. After stirring at room temperature overnight, water (50ml) was added and the product was extracted with Et_2_O (3 x 30ml). The combined organic extracts were dried over Na_2_SO_4_, filtered, concentrated under reduced pressure and purified by flash column chromatography to afford the products.

***Adamantyl-1,2,4-trioxolane MIS13.***

Prepared according to general procedure 2 to give **MIS13** as yellow oil. (41% yield). ^1^H-NMR (400 MHz, CDCl_3_): δ 1.25 (s, 1H), 1.43 (s, 11H), 1.49-1.50 (m, 4H), 1.67-1.82 (m, 14H), 1.89-1.95 (m, 8H), 3.11 (s, 2H), 3.25 (q, 2H) ppm; MS (MALDI-TOF), *m/z* 478.35 [M]^+^.

***Adamantyl-1,2,4-trioxolane LC92.***

Prepared according to general procedure 2 to give **LC92** as light yellow oil. (39% yield). ^1^H-NMR (400 MHz, CDCl_3_): δ 0.81-0.86 (m, 6H), 1.18-1.25 (m, 11H), 1.61-1.67 (m, 6H), 1.83-2.04 (m, 8H), 3.17 (dd, 2H) ppm; MS (MALDI-TOF), *m/z* 364.23 [M]^+^.

***Adamantyl-1,2,4-trioxolane LC142.***

Prepared according to general procedure 2 to give **LC142** as light yellow oil. (35% yield). ^1^H-NMR (400 MHz, CDCl_3_): δ 0.82-0.83 (m, 3H), 1.61-1.91 (m, 19H), 2.30 (m, 1H), 4.15 (s, 1H) ppm; MS (MALDI-TOF), *m/z* 414.34 [M+K]^+^.

***S.1.3.3. Preparation of trioxolanes MIS14, MIS16 and LC136***

**Scheme S6**: Reagents and conditions: i) LC64, AcOH, DCE, NaBH(OAc)_3_, r.t.; ii) Trichloroacetic acid, DCM, H_2_O, r.t.; iii) 5-Aminotetrazole monohydrate, AcOH, DCE, NaBH(OAc)_3_, r.t.

***General procedure 3: Preparation of adamantyl-1,2,4-trioxolanes MIS14 and LC136.*** The required amine (**LC64** or **5-aminotetrazole**) (3.4 mmol) was added to a solution of compound **LC50** (3.4 mmol), in anhydrous 1,2-dichloroethane (20 mL) and acetic acid (3.4 mmol). The mixture was allowed to stir at room temperature for 30 minutes, followed by addition of sodium triacetoxyborohydride (8.5 mmol). After stirring at room temperature for 16 hours, the final reaction mixture was washed with aqueous NaOH (5M; 2 x 10 mL) and dichloromethane (2 x 20 mL). The organic extract was dried over MgSO_4_, filtered, and the solvent evaporated. Purification of the crude by column chromatography (silica gel, ethyl acetate/n-hexane 3/7).

***Adamantyl-1,2,4-trioxolane MIS14.***

Prepared according to general procedure 3 to give **MIS14** as a brown oil (83% yield). ^1^H-NMR (400 MHz, CDCl_3_): δ 0.89 (m, 3H), 1.31 (m, 3H), 1.67 (m, 12H), 1.97 (m, 8H), 2.74 (td, 3H), 3.11 (s, 2H) ppm; MS (MALDI-TOF), *m/z* 451,32 [M+K]^+^.

***Adamantyl-1,2,4-trioxolanes LC136.***

Prepared according to general procedure 3 to give **LC136** as a white solid (80% yield); mp 98-100 ºC. ^1^H-NMR (400 MHz, CDCl_3_): δ 1.17 (t, 2H), 1.62-1.76 (m, 11H), 1.83-2.00 (m, 9H) ppm; MS (MALDI-TOF), *m/z* 384.26 [M+Cl]^-^.

***General Procedure 4: Preparation of adamantyl-1,2,4-trioxolanes MIS15 and MIS16.*** To a solution of **MIS13** or **MIS14** (1 eq.) in a solvent mixture of CH_2_Cl_2_ (26 mL) and water (0.05 mL) was added trifluoroacetic acid (10 mL), and the resulting solution was stirred at room temperature for 1 h. The solvent was subsequently evaporated to dryness under reduced pressure and the residue was purified by chromatography to afford the products.

***Adamantyl-1,2,4-trioxolane MIS15.***

Prepared according to general procedure 4 to give **MIS15** as orange oil. (87% yield). ^1^H-NMR (400 MHz, CDCl_3_): δ 1.17 (m, 1H), 1.21 (m, 2H), 1.22-1.34 (m, 4H), 1.77-1.79 (m, 14H), 1.89-1.97 (m, 8H), 3.43 (d, 2H), 4.06 (q, 2H) ppm; MS (MALDI-TOF), *m/z* 415.01 [M]^+^.

***Adamantyl-1,2,4-trioxolane MIS16.***

Prepared according to general procedure 4 to give **MIS16** as orange oil (98% yield). ^1^H-NMR (400 MHz, CDCl_3_): δ 0.85 (m, 3H), 1.18 (m, 3H), 1.21 (m, 2H), 1.34 (m, 3H), 2.05 (m, 8H), 2.44 (t, 4H), 3.67 (s, 2H) ppm; MS (MALDI-TOF), *m/z* 349,18 [M]^+^.

***S.1.4. Preparation of tetraoxanes***

The synthetic approach followed to tetraoxanes is depicted in Scheme S7. Synthetic procedures for each compound prepared are also provided in this section.

**Scheme S7**: Synthetic route to tetraoxanes LC140 and LC163. Reagents and conditions: i) HCO_2_H, CH_3_CN, H_2_O_2_ 50%, 0 ºC; ii) 1,4-cyclohexanone, DCM, HBF_4_, 0ºC; iii) DCE, AcOH, NaBH(OAc)_3_, r.t.

***Adamantyl-1,2,4,5-tetraoxane LC140.***

To a stirring solution of 2-adamantanone (5 mmol) in acetonitrile (5.5 mL) and formic acid (3.7 mL) at 0 ºC was added 50% aq. hydrogen peroxide (1.9 ml). The solution was allowed to warm to room temperature and stirred for 45 min. The solution was diluted with dichloromethane (100 ml) and washed with water (100 ml). The organic phase was dried over Na_2_SO_4_, filtered and concentrated to give the corresponding gem-dihydroperoxide. A solution of this intermediate in dichloromethane (5 mL) was added to a stirring solution of 1,4-cyclohexanone (7.5 mmol) and 54% ethereal solution of HBF_4_ (0.1 mL) in dichloromethane (5 mL) at 0ºC. The mixture was allowed to warm to room temperature and stirred for 4h. The organic layer was washed with a saturated solution of NaHCO_3_ and dried over MgSO_4_ and the solvent removed. The resulting residue was purified by flash column chromatography to give to give **LC140** as a white solid (50% yield); m.p. 156-158 ºC. ^1^H-NMR (400 MHz, CDCl_3_): δ 1.74 (s, 5H), 1.82-2.11 (m, 14H), 3.08 (t, 3H) ppm; MS (MALDI-TOF), *m/z* 318.28 [M+Na]^+^.

***Adamantyl-1,2,4,5-tetraoxane LC163.***

To a solution of compound **LC140** (3.4 mmol) in anhydrous 1,2-dichloroethane (20 mL) was added amino compounds (3.74 mmol) and acetic acid (3.4 mmol). The mixture was allowed to stir at room temperature for 30 minutes followed by addition of sodium triacetoxyborohydride (8.5 mmol). After stirring at room temperature for 16 hours, the reaction mixture was washed with aqueous NaOH (5M; 2 x 10 mL) and dichloromethane (2 x 20 mL). The organic extract was dried over MgSO_4_, filtered, and the solvent removed. The crude product was purified by column chromatography (silica gel, ethyl acetate/n-hexane 3/7) to give **LC163** as a white solid (95% yield); m.p. 142-144 ºC. ^1^H-NMR (400 MHz, CDCl_3_): δ 1.18 (t, 2H), 1.69-1.79 (m, 11H), 1.85-2.04 (m, 9H) ppm; MS (MALDI-TOF), *m/z* 399.17 [M+Cl]^-^.

**Table 1:** Antiplasmodial activity *in vitro* (IC_50_) against resistant and sensitive *P. falciparum* strains.

| **Compounds** | | ***P. falciparum* IC_50_ ± SD (nM)** | | | | |
| --- | --- | --- | --- | --- | --- | --- |
| **Reference** | **R1** | **Dd2** | **3D7** | **IPC5202** | **IPC4912** | **RI^a^** |
| **LC28** |  | 1127.0 ± 188.3 | 3659.0 ± 1488.2 | n.d. | n.d. | 0.3 |
| **LC50*** |  | 23.6 ± 8.4 | 14.0 ± 6.7 | n.d. | n.d. | 1.7 |
| **LC60** |  | > 10000 | >10000 | n.d. | n.d. | n.d. |
| **LC64** |  | > 10000 | >10000 | n.d. | n.d. | n.d. |
| **LC92** |  | 22.6 ± 2.6 | 26.7 ± 2.4 | 37.7 ± 9.9 | 34.9 ± 7.9 | 0.8 |
| **LC95** |  | 56.3 ± 1.0 | 29.55 ± 5.18 | n.d. | n.d. | 3.1 |
| **LC126II** |  | >10000 | >10000 | n.d. | n.d. | n.d. |
| **LC129** |  | 43.2 ± 0.8 | 42.9 ± 3.9 | 36.36 ± 1.2 | 29.3 ± 0.7 | 1.0 |
| **LC130** |  | 24.8 ± 17.2 | 23.3 ± 15.2 | 8.2 ± 1.9 | 2.4 ± 1.6 | 1.0 |
| **LC131** |  | 2.1 ± 1.9 | 2.9 ± 2.4 | 3.4 ± 2.1 | 4.8 ± 1.2 | 0.7 |
| **LC132** |  | 26.6 ± 15.9 | 24.2 ± 12.8 | 25.0 ± 7.4 | 19.7 ± 3.4 | 1.1 |
| **LC133** |  | >10000 | >10000 | n.d. | n.d. | n.d. |
| **LC136** |  | 2.5 ± 1.3 | 2.8 ± 1.2 | 6.1 ± 1.9 | 6.8 ± 2.3 | 0.9 |
| **LC142** |  | 106.9 ± 45.4 | 116.7 ± 33.2 | n.d. | n.d. | 0.9 |
| **LC154** |  | >10000 | >10000 | n.d. | n.d. | n.d. |
| **LC155** |  | >10000 | >10000 | n.d. | n.d. | n.d. |
| **MIS13** |  | 3.4 ± 3.0 | 7.4 ± 6.5 | 1.7 ± 1.1 | 0.3 ± 0.3 | 0.5 |
| **MIS14** |  | 44.6 ± 32.8 | 39.1 ± 12.5 | 413.1 ± 20.2 | 389.5 ± 17.8 | 1.1 |
| **MIS15** |  | ♦ | ♦ | ♦ | ♦ | n.d. |
| **MIS16** |  | ♦ | ♦ | ♦ | ♦ | n.d. |
| **LC163** |  | 43.5 ± 3.0 | 63.0 ± 0.7 | 71.1 ± 1.1 | 18.6 ± 16.9 | 0.7 |
| **ART** |  | 2.47 ± 0.1 | 3.97 ± 0.08 | 33.3 ± 8.0 | 13.3 ± 2.2 | 0.6 |
| **ATN** |  | 4.6 ± 1.4 | 5.1 ± 0.1 | 3.4 ± 0.1 | 3.9 ± 1.2 | 0.9 |
| **DHA** |  | 4.2 ± 0.5 | 4.7 ± 1.5 | 6.2 ± 1.6 | 3.7 ± 2.0 | 0.9 |
| **CQ** | 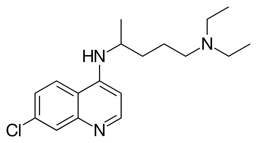 | 340.0 ± 20.7 | 15.8 ± 0.8 | n.d. | n.d. | 21.5 |

* Lobo et al, 2016

♦ Low solubility

n.d. - not determined.

ART: Artemisinin.

ATN: Artesunate.

DHA: Dihydroartemisinin.

CQ: Chloroquine.

^a^ RI (Resistance index) = IC_50_ (Dd2)/ IC_50_ (3D7).

**Table 2:** Selectivity index for compounds in V79 e HepG2 cell lines.

|  | **IS^a^ V79** | | | | | **IS^b^ HepG2** | | | | |
| --- | --- | --- | --- | --- | --- | --- | --- | --- | --- | --- |
| **Compound** | **LD_50_ (mM)** | **Dd2** | **3D7** | **IPC5202** | **IPC4912** | **LD_50_**  **(mM)** | **Dd2** | **3D7** | **IPC5202** | **IPC4912** |
| **LC28** | 1.0 | 887.3 | 273.3 | n.d. | n.d. | 1.0 | 887.3 | 273.3 | n.d. | n.d. |
| **LC50** | 1.0 | 42372.8 | 71428.6 | n.d. | n.d. | 1.0 | 42372.8 | 71428.6 | n.d. | n.d. |
| **LC60** | 1.0 | ♦ | ♦ | n.d. | n.d. | 1.0 | ♦ | ♦ | n.d. | n.d. |
| **LC64** | 1.0 | ♦ | ♦ | n.d. | n.d. | 1.0 | ♦ | ♦ | n.d. | n.d. |
| **LC92** | 1.0 | 44247.8 | 37453.1 | 26525.2 | 28653.3 | 1.0 | 44247.8 | 37453.1 | 26525.2 | 28653.3 |
| **LC95** | 1.0 | 1784.7 | 3441.7 | n.d. | n.d. | 1.0 | 1784.7 | 3441.7 | n.d. | n.d. |
| **LC126II** | 1.0 | ♦ | ♦ | n.d. | n.d. | 1.0 | ♦ | ♦ | n.d. | n.d. |
| **LC129** | 1.0 | 23148.1 | 23310.0 | 27502.7 | 29673.5 | 1.0 | 23148.1 | 23310.0 | 27502.7 | 29673.5 |
| **LC130** | 1.0 | 40322.6 | 42918.4 | 121951.2 | 416666.6 | 1.0 | 40322.6 | 42918.4 | 121951.2 | 416666.6 |
| **LC131** | 1.0 | 476190.5 | 344827.5 | 294117.6 | 208333.3 | 1.0 | 476190.5 | 344827.5 | 294117.6 | 208333.3 |
| **LC132** | 1.0 | 37596.9 | 41322.3 | 40000.0 | 50761.4 | 1.0 | 37596.9 | 41322.3 | 40000.0 | 5076.4 |
| **LC133** | 1.0 | ♦ | ♦ | n.d. | n.d. | 1.0 | ♦ | ♦ | n.d. | n.d. |
| **LC136** | 1.0 | 400000.0 | 357142.8 | 163934.4 | 147058.8 | 1.0 | 400000.0 | 357142.8 | 163934.4 | 147058.8 |
| **LC142** | 1.0 | 9354.5 | 8568.9 | n.d. | n.d. | 1.0 | 9354.5 | 8568.9 | n.d. | n.d. |
| **LC154** | 1.0 | ♦ | ♦ | n.d. | n.d. | 1.0 | ♦ | ♦ | n.d. | n.d. |
| **LC155** | 1.0 | ♦ | ♦ | n.d. | n.d. | 1.0 | ♦ | ♦ | n.d. | n.d. |
| **MIS13** | 1.0 | 294117.6 | 135135.1 | 588235.2 | 3333333.3 | 0.3 | 97058.8 | 44594.6 | 194117.6 | 1100000.0 |
| **MIS14** | 1.0 | 22421.5 | 25575.4 | 2420.7 | 2567.4 | 0.3 | 7399.1 | 8439.9 | 798.8 | 847.2 |
| **MIS15** | 1.0 | ♦ | ♦ | n.d. | n.d. | 1.0 | ♦ | ♦ | n.d. | n.d. |
| **MIS16** | 1.0 | ♦ | ♦ | n.d. | n.d. | 1.0 | ♦ | ♦ | n.d. | n.d. |
| **LC163** | 1.0 | 22988.5 | 15873.0 | 14064.7 | 53763.4 | 1.0 | 22988.5 | 15873.0 | 14064.7 | 53763.4 |
| **ART** | 0.4 | 168515.4 | 104844.6 | 12499.5 | 31295.7 | 0.4 | 168515.4 | 104844.6 | 12499.5 | 31295.7 |
| **DHA** | 1.0 | 238095.2 | 212765.9 | 166666.7 | 270270.3 | 1.0 | 238095.2 | 212765.9 | 166666.7 | 270270.3 |
| **CQ** | 0.2 | 588.2 | 12658.2 | n.d. | n.d. | 0.2 | 588.2 | 12658.2 | n.d. | n.d. |

^a^ SI (Selectivity index) = LD_50_ (V79)/ IC_50_ (Dd2, 3D7, IPC5202 and IPC4912)

^b^ SI (Selectivity index) = LD_50_ (HepG2)/ IC_50_ (Dd2, 3D7, IPC5202 and IPC4912)

♦ Low solubility

n.d. - not determined.
